# Dietary glutamic acid and aspartic acid as biomarkers for predicting diabetic retinopathy

**DOI:** 10.1038/s41598-021-83165-5

**Published:** 2021-03-31

**Authors:** So Young Park, Jieun Kim, Jung Il Son, Sang Youl Rhee, Do-Yeon Kim, Suk Chon, Hyunjung Lim, Jeong-Taek Woo

**Affiliations:** 1 Department of Endocrinology & Metabolism, Kyung Hee University Hospital, Seoul, Republic of Korea; 2 Research Institute of Medical Nutrition, Kyung Hee University, Seoul, Republic of Korea; 3 Il San Gospel Hospital, Goyang-si, Gyeonggi-do, Republic of Korea; 4 Department of Endocrinology and Metabolism, Kyung Hee University School of Medicine, 23 Kyungheedae-ro, Dongdaemun-gu, Seoul, 02447 Republic of Korea; 5 Department of Medical Nutrition, Graduate School of East-West Medical Science, Kyung Hee University, 1732 Deogyeong-daero, Giheung-gu, Yongin, 17104 Republic of Korea

**Keywords:** Biomarkers, Endocrinology

## Abstract

The screening rate of diabetic retinopathy (DR) is low despite the importance of early diagnosis. We investigated the predictive value of dietary glutamic acid and aspartic acid for diagnosis of DR using the Korea National Diabetes Program cohort study. The 2067 patients with type 2 diabetes without DR were included. The baseline intakes of energy, glutamic acid and aspartic acid were assessed using a 3-day food records. The risk of DR incidence based on intake of glutamic acid and aspartic acid was analyzed. The DR group was older, and had higher HbA1c, longer DM duration, lower education level and income than non-DR group (all *p* < 0.05). The intake of total energy, glutamic acid and aspartic acid were lower in DR group than non-DR group (*p* = 0.010, *p* = 0.025 and *p* = 0.042, respectively). There was no difference in the risk of developing DR according to the intake of glutamic acid and ascorbic acid. But, aspartic acid intake had a negative correlation with PDR. Hence, the intake of glutamic acid and aspartic acid did not affect in DR incidence. However, lower aspartic acid intake affected the PDR incidence.

## Introduction

Diabetic retinopathy (DR) is a common microvascular complication associated with diabetes. The reported global prevalence of DR in type 2 diabetes mellitus (T2DM) patients is 30–40%^1^. Because DR is more specific to hyperglycemia than other diabetic complications, it is referenced in the diagnosis of DM^2^. Chronic hyperglycemia induced retinal vascular endothelial dysfunction causes retinal ischemia and increased vascular permeability, resulting in vision-threatening DR^3^. Moreover, DR accounts for 4.8% of blindness^4^. And as diabetes prevalence increases worldwide, DR is considered a leading cause of visual loss^5^. Visual impairment due to DR can not only reduce patient quality of life but also increase medical expenses and result in substantial burden to the national healthcare system. While early detect and proper management of DR can prevent severe vision loss or blindness^3^, the screening rate for DR is significantly lower than that for other complications^6^ because the specific instrument such as fundus camera and skilled physician are required for screening^7^. Therefore, previous studies have sought to identify effective biomarkers easier screening for DR as well as predicting treatment response and prognosis. However, the biomarkers already used widely in clinical research and practice lack accurate prediction; thus, residual risk remains^8^.

Our recent metabolomics study in a well-organized cohort of geriatric diabetic patients showed that some important metabolites such as plasma glutamic acid, and aspartic acid, etc. were directly related to DR^9^. However, the study was cross-sectional in design; thus, it was difficult to identify causal relationships. Therefore, we sought to obtain more clear evidence using large-scale, multicenter, prospective cohort data. This study aimed to investigate the causal relationship between intakes of glutamic and aspartic acids and DR based on an analysis of large-scale, multicenter, prospective cohort data. We also classified non-proliferative diabetic retinopathy (NPDR) and proliferative diabetic retinopathy (PDR) according to DR severity and analyzed the relationship between DR severity and dietary glutamic acid and aspartic acid.

## Results

### Baseline subject characteristics

**Figure 1:**
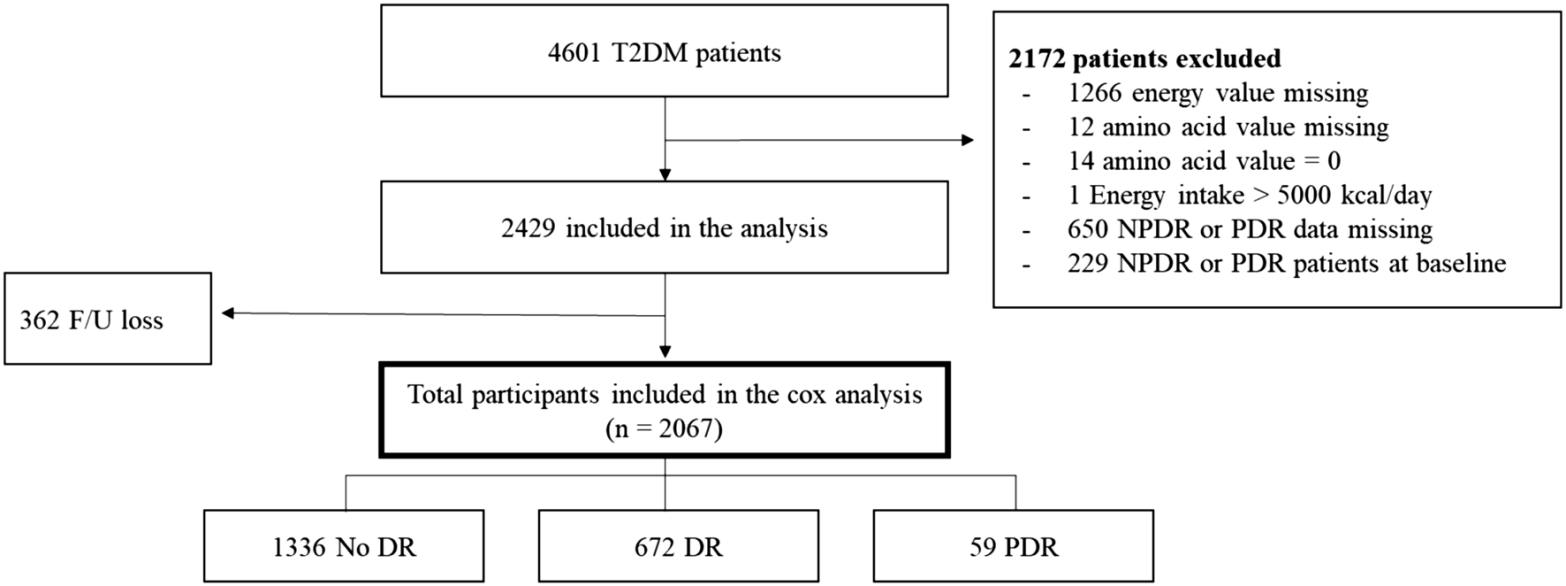
Study progression. *DM* diabetes mellitus; *NPDR* non-proliferative diabetic retinopathy; *PDR* proliferative diabetic retinopathy; *DR* diabetic retinopathy.

Of the 2429 subjects recruited to the study, 2067 completed the study, including 731 (35.4%) diagnosed with DR; 672 (32.5%) with NPDR, and 59 (2.9%) with PDR (Fig. 1).

**Figure 2:**
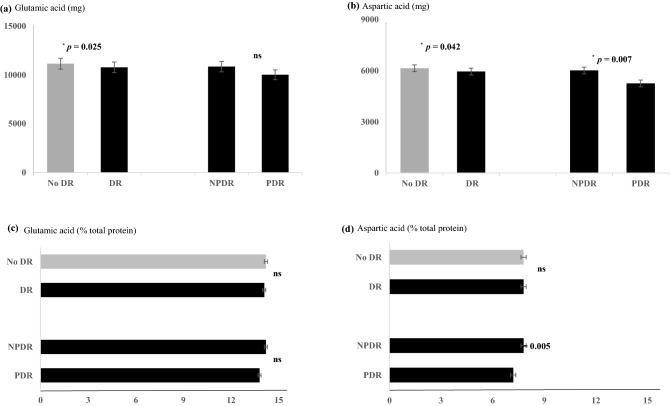
The differences of glutamic acid and aspartic acid intakes (mg or % total protein). The glutamic acid (**a**) and aspartic acid intakes (**b**) were lower in the DR group than those in the non-DR. There was no difference in proportion of total protein of glutamic acid (**c**) and aspartic acid (**d**). The absolute intake (**b**) and proportion of total protein (**d**) of aspartic acid were lower in the PDR group than those in the NPDR group. By general linear model (GLM), *ns* non-significant. *NPDR* non-proliferative diabetic retinopathy; *PDR* proliferative diabetic retinopathy; *DR* diabetic retinopathy

The baseline characteristics of the subjects by DR status are shown in Table 1. The DR group was older (*p* < 0.0001), and had a higher glycated hemoglobin (HbA1c) (*p* < 0.0001), longer DM duration (*p* < 0.0001), lower education level (*p* = 0.011), and lower income (*p* = 0.001) than those in the non-DR group. Although all values were within the normal ranges, the alanine aminotransferase (ALT) was lower (*p* = 0.007), and blood urea nitrogen (BUN) were higher (*p* = 0.046) in the DR group than those in the non-DR group. The low-density lipoprotein (LDL) cholesterol level was higher and the high-density lipoprotein (HDL) cholesterol level was lower in the DR group (*p* = 0.013 and *p* = 0.025, respectively). The DR group had a higher proportion of participants with a history of hypertension (HTN) and cerebrovascular disease (*p* = 0.004 and *p* = 0.002, respectively). The total energy, glutamic acid, and aspartic acid intakes were lower in the DR group than those in the non-DR group (*p* = 0.010, *p* = 0.025, and *p* = 0.042, respectively) (Fig. 2). Among subjects with DR, no difference in HbA1c was observed between the NPDR and PDR groups; however, the PDR group had a longer DM duration than that in the NPDR group. Renal dysfunctions and lower aspartic acid intake were observed in the PDR group compared to those in the NPDR group.

**Table 1:** Baseline characteristics of the study subjects according to DR status.

	No DR (n = 1336)	DR (n = 731)				*p* value (no DR vs DR)
		NPDR (n = 672)	PDR (n = 59)	*p* value (NPDR vs PDR)	DR (n = 731)	
Age (years)	53.1 ± 9.7	55.5 ± 9.6	56.1 ± 11.0	0.678	55.6 ± 9.7	< 0.0001
Gender (n, %)						0.245
Men	781 (58.5)	375 (55.8)	33 (55.9)	0.985	408 (55.8)	
Women	555 (41.5)	297 (44.2)	26 (44.1)		323 (44.2)	
DM duration (years)	5.5 ± 5.5	7.7 ± 6.7	11.9 ± 8.6	0.008	8.0 ± 7.0	< 0.0001
Education (n, %)						
Middle school ≤	433 (34.3)	257 (39.1)	23 (41.1)	0.329	280 (39.2)	0.011
High school	451 (35.7)	239 (36.3)	24 (42.9)		263 (36.8)	
College/University	378 (30.0)	162 (24.6)	9 (16.1)		171 (24.0)	
Income, won/month (n, %)						0.001
≤ 200 × 10^4^	433 (35.9)	268 (42.7)	27 (50.9)	0.208	295 (43.3)	
< 201 × 10^4^ and ≤ 400 × 10^4^	412 (34.2)	212 (33.8)	19 (35.9)		231 (33.9)	
> 401 × 10^4^	360 (29.9)	148 (23.4)	7 (13.2)		155 (22.8)	
Occupation (n, %)				0.005		0.423
Administration, office workers	234 (18.7)	108 (16.5)	5 (9.3)		113 (16.0)	
Sales, service, agricultural, laborer, piscatorial	276 (22.1)	133 (20.3)	15 (27.8)		148 (20.9)	
Self-employed	169 (13.5)	94 (14.4)	4 (7.4)		98 (13.8)	
Homemaker	379 (30.3)	106 (16.2)	18 (33.3)		225 (31.8)	
Student, not employed, army	192 (15.4)	213 (32.6)	12 (22.2)		124 (17.5)	
Smoking (n, %)				0.523		0.055
Current	302 (22.7)	131 (19.5)	9 (15.3)		140 (19.2)	
Ex-smoker	380 (28.5)	181 (27.0)	14 (23.7)		195 (26.7)	
Never	651 (48.8)	359 (53.5)	36 (61.0)		395 (54.1)	
Alcohol (n, %)				0.547		0.057
Current	702 (52.7)	320 (47.8)	26 (44.1)		346 (47.5)	
Ex-drinker	128 (9.6)	62 (9.3)	8 (13.6)		70 (9.6)	
Never	503 (37.7)	288 (43.0)	25 (42.4)		313 (42.9)	
BMI (kg/m^2^)	25.2 ± 0.1	25.0 ± 0.1	24.9 ± 0.4	0.648	25.0 ± 0.1	0.345
SBP (mmHg)	124.8 ± 0.4	125.3 ± 0.6	128.5 ± 2.0	0.112	125.3 ± 0.5	0.454
DBP (mmHg)	78.1 ± 0.3	77.6 ± 0.4	77.9 ± 1.3	0.810	77.8 ± 0.4	0.498
HbA1c (%)	7.6 ± 0.0	8.0 ± 0.1	8.2 ± 0.2	0.364	8.0 ± 0.0	< 0.0001
TC (mg/dL)	178.6 ± 1.1	181.6 ± 1.6	182.8 ± 5.5	0.833	182.1 ± 1.5	0.063
TG (mg/dL)	159.3 ± 3.4	157.4 ± 4.5	137.2 ± 15.8	0.222	158.2 ± 4.6	0.837
HDL-C (mg/dL)	48.3 ± 0.4	47.0 ± 0.5	47.1 ± 1.8	0.962	46.9 ± 0.5	0.025
LDL-C (mg/dL)	99.8 ± 1.0	103.8 ± 1.5	106.3 ± 5.3	0.661	104.2 ± 1.4	0.013
BUN (mg/dL)	14.6 ± 0.1	15.1 ± 0.2	15.5 ± 0.7	0.530	15.0 ± 0.2	0.046
Creatinine (mg/dL)	0.8 ± 0.0	0.8 ± 0.0	0.9 ± 0.0	0.016	0.8 ± 0.0	0.321
CrCl (mL/min)	94.5 ± 0.8	94.9 ± 1.2	85.1 ± 4.1	0.020	96.6 ± 1.0	0.113
AST (IU/l)	26.1 ± 0.4	25.0 ± 0.5	23.3 ± 1.8	0.374	25.0 ± 0.5	0.094
ALT (IU/l)	30.2 ± 0.6	27.5 ± 0.7	25.9 ± 2.5	0.546	28.0 ± 0.8	0.034
Comorbidity (n, %)						
Hypertension	618 (46.3)	360 (53.7)	34 (57.6)	0.724	394 (53.0)	0.004
Dyslipidemia	591 (44.2)	289 (43.1)	25 (42.4)	0.974	314 (43.0)	0.808
Cardiovascular disease	72 (5.4)	45 (6.8)	7 (11.9)	0.148	52 (7.2)	0.110
Cerebrovascular disease	50 (3.8)	48 (7.2)	2 (3.4)	0.266	50 (6.9)	0.002
Dietary intake						
Energy (kcal)	1816.6 ± 375.9	1,774.2 ± 377.5	1,741.7 ± 403.5	0.529	1771.6 ± 379.5	0.010
Glutamic acid (mg)	11,149.0 ± 3594.5	10,851.5 ± 3488.5	10,011.9 ± 3127.2	0.074	10,783.7 ± 3466.3	0.025
Glutamic acid (% total protein)	14.2 ± 2.5	14.2 ± 2.5	13.8 ± 3.1	0.241	14.1 ± 2.5	0.315
Aspartic acid (mg)	6142.0 ± 2072.7	6009.9 ± 2052.6	5252.8 ± 1669.9	0.007	5948.8 ± 2034.0	0.042
Aspartic acid (% total protein)	7.8 ± 1.6	7.8 ± 1.5	7.2 ± 1.7	0.005	7.8 ± 1.6	0.395

Data shown as mean ± SD or %, by Chi-squared test or general linear model (GLM).

*DR* diabetic retinopathy; *NPDR* isolated non-proliferative diabetic retinopathy; *PDR* proliferative diabetic retinopathy, *BMI* body mass index; *SBP* systolic blood pressure; *DBP* diastolic blood pressure, *HbA1c* glycated hemoglobin; *TC* total cholesterol; *TG* triglyceride; *HDL-C* high density lipoprotein cholesterol; *LDL-C* low density lipoprotein cholesterol; *BUN* blood urea nitrogen; *CrCl* creatinine clearance; *AST* aspartate aminotransferase; *ALT* alanine aminotransferase.

### The effect of dietary glutamic and aspartic acid on DR incidence

The median follow-up period was 5 years. The incidence of DR was 210.4 cases/1000 person/year; NPDR, 193.4 cases/1000 person/year; and PDR, 17.0 cases/1000 person/year (Table 2).

**Table 2:** Incidence rate of DR.

	Total (n = 2067)	DR		
		Total DR (n = 731)	NPDR (n = 672)	PDR (n = 59)
Median follow-up period, year	5.0	5.3	5.4	5.3
Case/1000 Person-years (PY)*		210.4	193.4	17.0

*DR* diabetic retinopathy; *NPDR* isolated non-proliferative diabetic retinopathy; *PDR* proliferative diabetic retinopathy.

*PY: Person year, By Miettinen's (1974d) modification, as described in Epidemiologic Analysis with a Programmable Calculator, 1979. Results from OpenEpi, Version 3, open source calculator–PersonTime1.

The absolute intakes and proportions of total protein intake of glutamic acid and aspartic acid were divided into tertiles and the DR risk according to tertiles of absolute intake of glutamic acid and aspartic acid was analyzed (Table 3). The hazard ratio (HR) of glutamic acid for DR was 1.00 [95% confidence interval (CI) 0.78–1.28] in the middle tertile, and 0.90 (95% CI 0.66–1.22) in the highest tertile compared to the lowest tertile. The HR of aspartic acid for DR was 1.04 (95% CI 0.81–1.34) in the middle tertile, and 0.90 (95% CI 0.66–1.23) in the highest tertile compared to the lowest tertile. After adjusting for confounding factors, no difference in DR risk was observed according to tertiles of absolute intake (mg) of glutamic acid and aspartic acid. However, the absolute intake of aspartic acid was associated with PDR. The HR of aspartic acid for PDR was 0.12 (95% CI 0.02–0.98) in the highest tertile compared to the lowest tertile. The results remained consistent after adjusting for confounding factors. Table 4 shows the DR risk according to tertiles of the proportions of total protein intake of glutamic and aspartic acids. These proportions of total protein intake also did not affect the risk of DR. However, the negative effect of aspartic acid on PDR was more pronounced. The HR of aspartic acid for PDR was 0.43 (95% CI 0.19–0.94) in the middle tertile, and 0.43 (95% CI 0.20–0.94) in the highest tertile compared to the lowest tertile. The results remained consistent after adjustment.

**Table 3:** Hazard ratios for diabetic retinopathy incidence according to tertiles of glutamic acid and aspartic acid intake (mg).

	Tertiles of glutamic acid (mg)			*p for trend*	Tertiles of aspartic acid (mg)			*p for trend*
	T1	T2	T3		T1	T2	T3	
**DR**								
HR (95% CI)								
Crude	1 [ref]	1.00 (0.78–1.28)	0.90 (0.66–1.22)	0.436	1 [ref]	1.04 (0.81–1.34)	0.90 (0.66–1.23)	0.411
Model 1	1 [ref]	1.03 (0.80–1.33)	0.97 (0.70–1.34)	0.808	1 [ref]	1.08 (0.84–1.39)	0.96 (0.70–1.33)	0.724
Model 2	1 [ref]	1.02 (0.79–1.24)	1.01 (0.82–1.24)	0.617	1 [ref]	1.07 (0.83–1.39)	0.93 (0.67–1.29)	0.551
Model 3	1 [ref]	0.95 (0.72–1.24)	0.91 (0.65–1.28)	0.593	1 [ref]	1.01 (0.77–1.32)	0.89 (0.64–1.25)	0.442
Model 4	1 [ref]	0.97 (0.73–1.28)	0.94 (0.66–1.33)	0.711	1 [ref]	1.01 (0.77–1.33)	0.88 (0.62–1.25)	0.427
**NPDR**								
HR (95% CI)								
Crude	1 [ref]	1.00 (0.77–1.29)	0.94 (0.69–1.29)	0.681	1 [ref]	1.04 (0.80–1.35)	0.97 (0.71–1.34)	0.794
Model 1	1 [ref]	1.03 (0.80–1.34)	1.03 (0.74–1.43)	0.875	1 [ref]	1.09 (0.84–1.41)	1.06 (0.76–1.47)	0.806
Model 2	1 [ref]	1.02 (0.83–1.30)	0.99 (0.71–1.39)	0.942	1 [ref]	1.08 (0.82–1.41)	1.03 (0.74–1.43)	0.956
Model 3	1 [ref]	0.96 (0.73–1.27)	0.99 (0.70–1.40)	0.985	1 [ref]	1.03 (0.78–1.36)	1.00 (0.71–1.42)	0.982
Model 4	1 [ref]	0.97 (0.73–1.30)	1.01 (0.71–1.45)	0.901	1 [ref]	1.01 (0.76–1.35)	0.98 (0.68–1.39)	0.859
**PDR**								
HR (95% CI)								
Crude	1 [ref]	1.11 (0.46–2.67)	0.43 (0.11–1.74)	0.233	1 [ref]	0.98 (0.43–2.22)	0.12 (0.02–0.98)	**0.036**
Model 1	1 [ref]	1.07 (0.44–2.61)	0.41 (0.10–1.67)	0.188	1 [ref]	0.94 (0.41–2.15)	0.11 (0.01–0.91)	**0.028**
Model 2	1 [ref]	1.10 (0.45–2.68)	0.39 (0.10–1.64)	0.553	1 [ref]	1.01 (0.44–2.35)	0.12 (0.01–0.96)	**0.035**
Model 3	1 [ref]	0.90 (0.36–2.27)	0.28 (0.06–1.25)	0.083	1 [ref]	0.83 (0.35–2.01)	0.09 (0.01–0.79)	**0.015**
Model 4	1 [ref]	0.93 (0.34–2.56)	0.18 (0.03–1.14)	0.059	1 [ref]	0.79 (0.31–2.06)	0.10 (0.01–0.90)	**0.024**

By Cox proportional hazard models.

DR, diabetic retinopathy; NPDR, isolated non-proliferative diabetic retinopathy; PDR, proliferative diabetic retinopathy.

Model 1: adjusted for age and sex, Model 2: adjusted for Model 1 + HbA1C, duration of diabetes,

Model 3: adjusted for Model 2 + education, income, occupation, Model 4: adjusted for Model 3 + CrCl, ALT, comorbidity.

**Table 4:** Hazard ratio for diabetic retinopathy incidence according to tertiles of glutamic acid and aspartic acid intake (% total protein).

	Tertiles of glutamic acid (% total protein)			*p for trend*	Tertiles of aspartic acid (% total protein)			*P for trend*
	T1	T2	T3		T1	T2	T3	
**DR**								
HR (95% CI)								
Crude	1 [ref]	0.99 (0.80–1.22)	0.96 (0.77–1.19)	0.704	1 [ref]	0.96 (0.78–1.19)	0.968 (0.78–1.20)	0.750
Model 1	1 [ref]	1.00 (0.81–1.23)	0.96 (0.77–1.19)	0.733	1 [ref]	0.95 (0.77–1.18)	0.970 (0.78–1.20)	0.758
Model 2	1 [ref]	1.00 (0.81–1.24)	0.96 (0.77–1.20)	0.762	1 [ref]	0.99 (0.80–1.23)	1.021 (0.82–1.27)	0.864
Model 3	1 [ref]	0.98 (0.79–1.23)	0.89 (0.71–1.12)	0.362	1 [ref]	0.99 (0.79–1.25)	0.995 (0.79–1.25)	0.962
Model 4	1 [ref]	0.99 (0.78–1.24)	0.89 (0.70–1.13)	0.368	1 [ref]	0.94 (0.75–1.19)	0.963 (0.76–1.22)	0.728
**NPDR**								
HR (95% CI)								
Crude	1 [ref]	1.03 (0.82–1.28)	0.99 (0.79–1.23)	0.922	1 [ref]	1.04 (0.83–1.30)	1.03 (0.83–1.29)	0.757
Model 1	1 [ref]	1.03 (0.83–1.29)	0.99 (0.79–1.24)	0.964	1 [ref]	1.03 (0.83–1.29)	1.04 (0.83–1.30)	0.731
Model 2	1 [ref]	1.04 (0.83–1.30)	1.00 (0.80–1.25)	0.971	1 [ref]	1.08 (0.86–1.35)	1.10 (0.88–1.37)	0.410
Model 3	1 [ref]	1.03 (0.82–1.29)	0.93 (0.73–1.19)	0.624	1 [ref]	1.08 (0.85–1.37)	1.08 (0.85–1.36)	0.526
Model 4	1 [ref]	1.04 (0.82–1.32)	0.95 (0.74–1.21)	0.711		1.04 (0.82–1.32)	1.05 (0.82–1.33)	0.711
**PDR**								
HR (95% CI)								
Crude	1 [ref]	0.68 (0.31–1.48)	0.77 (0.36–1.64)	0.434	1 [ref]	**0.43 (0.19–0.94)**	**0.43 (0.20–0.94)**	**0.017**
Model 1	1 [ref]	0.68 (0.31–1.47)	0.76 (0.35–1.62)	0.411	1 [ref]	**0.42 (0.19–0.92)**	**0.42 (0.19–0.93)**	**0.015**
Model 2	1 [ref]	0.72 (0.33–1.57)	0.75 (0.35–1.61)	0.416	1 [ref]	0.46 (0.21–1.02)	**0.46 (0.21–1.02)**	**0.031**
Model 3	1 [ref]	0.63 (0.27–1.47)	0.67 (0.29–1.51)	0.283	1 [ref]	**0.38 (0.16–0.92)**	**0.44 (0.19–1.03)**	**0.027**
Model 4	1 [ref]	0.55 (0.22–1.34)	0.47 (0.19–1.20)	0.091	1 [ref]	**0.26 (0.09–0.70)**	**0.39 (0.16–0.96)**	**0.013**

By Cox proportional hazard models.

*DR* diabetic retinopathy; *NPDR* isolated non-proliferative diabetic retinopathy; *PDR* proliferative diabetic retinopathy.

*Model 1, adjusted for age and sex.

**Model 2, adjusted for Model 1 + HbA1C, duration of diabetes mellitus.

***Model 3, adjusted for Model 2 + education, income, occupation.

****Model 4, adjusted for Model 3 + CrCl, ALT, comorbidity.

## Discussion

In our study, the patients with incident DR were older and had a higher HbA1c level, longer DM duration, higher LDL cholesterol level, and lower HDL cholesterol level than those in patients without incident DR. Additionally, more patients in the DR group had a history of HTN and cerebrovascular diseases. Uncontrolled DM, HTN, dyslipidemia, and long diabetes duration are well-known risk factors for DR^1,13^. In the present study, more patients in the DR group had cerebrovascular disease compared to the non-DR group, but no difference in cardiovascular disease was observed. Previous studies reported increased risks of cardiovascular disease in patients with DR^14,15^. However, as cerebrovascular disease better reflects microangiopathy involving small vessel diseases than cardiovascular disease^16^, more patients with DR might have cerebrovascular disease. The lower educational and economic state in the DR group may be associated with low availability of medical services.

Our previous cross-sectional study demonstrated plasma glutamine, glutamic acid, and their ratio as predictors of DR^9^. High plasma glutamine level and glutamine/glutamic acid ratio and low plasma glutamic acid level were associated with DR. In this study, the baseline intakes of energy, glutamic acid, and aspartic acid were lower in the DR group than those in the non-DR group. However, when divided into tertiles according to glutamic acid and aspartic acid intakes, we observed no difference in the risk of DR incidence. Whereas, a low intake of aspartic acid was associated with a higher risk of developing PDR. While PDR incidence tended to increase with low glutamic acid intake, the difference was not statistically significant.

The pathogenesis of DR is mainly retinal vascular change. Chronic hyperglycemia induces basement membrane thickening, pericyte loss, and microvascular aneurysm or occlusion, resulting in a pathologic change of the blood-retinal barrier. The resultant retinal ischemia and increased vascular permeability cause vision-threatening DR^17^. The biochemical mechanisms involve the accumulation of sorbitol and advanced glycation end products (AGE), oxidative stress, protein kinase C activation, inflammation, and upregulation of the renin-angiotensin system (RAS) and vascular endothelial growth factor (VEGF)^3^. Recently, not only retinal vascular changes but also neuronal damage are thought to be important due to DR pathogenesis^18^. Although the mechanism of retinal neuron changes in DR is not yet understood, hypoxia-induced retinal neuron alteration might secondarily affect retinal tissue.

The role of dietary glutamic and aspartic acids in DR is not yet known. Although glutamic acid is the major excitatory neurotransmitter, excessive extracellular accumulation of glutamic acid damages neurons through excitotoxic mechanisms^19^. Glutamic neurotoxicity could induce the death of retinal ganglion cells (RGCs) in DR or other ischemia-related ocular diseases^20,21^. Increased ROS production in the retina due to oxidative stress induced by hyperglycemia may impair glutamate-aspartate transporter (GLAST) function in Müller cells^22^. Müller cells regulate glutamate clearance in the retina through GLAST^22^. The level of asparagine and glutamine was increased in the aqueous humor of patients with DR, and highly activated alanine, aspartate, and glutamate metabolic pathway were identified in^1^H-NMR-based metabolomics analysis^23^.

Previous studies have shown the association of glutamic and aspartic acids with insulin resistance and secretion. The positive association between glutamic acid and waist circumference^24,25^ and visceral adipose tissue^26^ could be explained by the metabolism of branched-chain-amino-acids (BCAAs) in adipocytes to generate glutamic acid^27^. These findings suggest that glutamic acid was associated with increased insulin resistance, a finding consistent with reports in other studies that elevated plasma glutamic acid levels were associated with insulin resistance and abnormal fasting or 2-h glucose levels^24,28^. Moreover, a recent population-based large cohort study reported that nine amino acids, including glutamic acid and aspartic acid, were associated with decreased insulin secretion and elevated glucose level^29^. Increased insulin resistance and decreased insulin secretion can adversely affect blood glucose level, blood pressure, and blood lipid levels, increasing the risk of developing DR. However, the relationship between blood and retinal concentrations of glutamic and aspartic acids has not yet been reported.

In our study, the intakes of glutamic acid and aspartic acid did not affect the incidence of DR. This finding may be explained by the fact that the intake amount may not absolutely reflect blood levels. As non-essential amino acids, both glutamic acid and aspartic acid can be synthesized in the human body. Therefore, the blood levels of glutamic and aspartic acids are associated with both biosynthesis and dietary intake. Thus, the blood concentrations of glutamic and aspartic acids may be determined by biosynthesis in the body rather than intake.

Although glutamic acid and aspartic acid intakes did not affect DR development, aspartic acid intake affected DR severity, in which a lower intake was associated with PDR.

Alterations of some metabolites-related pathways in DR have been reported^30^. Because these alterations were related to the alanine-asparatic acid pathway and aspartic acid-asparagine pathway^30^, the influence of aspartic acid appears to be stronger.

This study has some limitations. First, dietary data on glutamic acid and aspartic acid intake were collected from dietary diaries. It is difficult to accurately extract and measure each dietary component in practice However, in this study, a trained dietitian used CAN Program for nutrient analysis. The CAN Program is widely using dietary assessment program in nutritional science in Korea. Second, the risk of developing DR was analyzed only from baseline intakes of glutamic and aspartic acids; however, whether baseline intake is representative of total intakes remains unknown. Therefore, dietary assessments were conducted every 3 years in this study, with no difference in the intakes of glutamic and aspartic acids compared to those at baseline. Recent studies have assessed the relationship between DR and metabolites. To our knowledge, the present study is the first to analyze the association of DR and metabolites absorbed by dietary intake.

In conclusion, the dietary glutamic acid and aspartic acid did not affect the incidence of DR. However, aspartic acid intake was negatively correlated with PDR. The metabolites absorbed by dietary intake may not directly affect the concentrations in blood and body fluids.

## Methods

The data for this study were derived from the Korea National Diabetes Program (KNDP) cohort study performed at 12 hospitals. The KNDP is a prospective, multicenter, observational study evaluating Korean patients with T2DM (NCT 01212198). The first patients were enrolled in May 2006 and the follow-up period was defined as the time between baseline and March 2014. Detailed descriptions of the KNDP cohort study design and data collection have been described elsewhere^10^.

Among 4601 T2DM patients, we excluded those with missing values in their dietary history (1266 energy values and 12 amino acid values) and NPDR and PDR data (n = 650). Participants with total energy intake outside the limits (< 500 and > 5000 kcal/day) (n = 1) and with amino acid levels that were unlikely to be accurate (n = 14) were also excluded. Participants who had been diagnosed with DR before the study period began were also excluded (n = 229). After applying these exclusion criteria, this study included 2429 subjects (1189 men, 878 women), of whom 2067 completed the study (Fig. 1).

This was a multicenter, prospective cohort study. The primary outcome was the incidence of DR during the follow-up period. The baseline data were collected based on sociodemographic (age, sex, education level, income, occupation, and smoking and alcohol consumption), clinical characteristics (body mass index, blood pressure), biochemical examination (glycemic control, lipid control, renal function, and liver function), DM duration, medical history (hypertension [HTN], dyslipidemia, cardiovascular or cerebrovascular disease), and dietary intakes. Dietary assessment was performed by a trained dietitian using a 3-day food records. The baseline energy, glutamic acid, and aspartic acid intakes were assessed and the absolute intake (mg) and proportion of total protein (%) determined. Nutrient analysis was performed using Computer-Aided Nutritional (CAN) analysis version 3.0 (Korean Nutrition Society, Seoul, South Korea). Dietary assessment was conducted every 3 years. DR was assessed by color fundus photography (FF 540 Plus; Carl Zeiss Meditech, Jena, Germany) and optical coherence tomography (HD-OCT; Carl Zeiss Meditech, Dublin, CA, USA) at baseline. All subjects underwent DR assessment annually. In accordance with Early Treatment Diabetic Retinopathy Study (ETDRS) criteria, DR was graded into three categories: non-DR, non-proliferative diabetic retinopathy (NPDR), or PDR^11,12^. Two or more ophthalmologists classified the DR status based on the examination results. Discordance between the evaluators was resolved by a review of the images to agree on the final interpretation.

The effects of glutamic acid and aspartic acid intake on DR incidence were analyzed by dividing the intake amount (mg or % protein) into tertiles. The HR for DR incidence in the middle and highest tertiles were compared to the lowest tertile.

The baseline characteristics of the subjects in the three groups (no DR, NPDR, PDR) are expressed as means ± standard deviations (SD) for continuous variables and as percentages for categorical variables. To compare the characteristics among groups, general linear models (GLMs) were used for continuous variables and chi-squared tests for categorical variables. The HRs and 95% confidence interval (CIs) for incident NPDR, PDR, and DR were calculated according to tertiles of glutamic and aspartic acid intakes (mg or % protein) by Cox proportional hazard models. In the multivariate-adjusted models, Model 1 was adjusted for age and sex, while Model 2 was adjusted for the covariates in Model 1 plus glycated hemoglobin (HbA1c) level, and DM duration. Model 3 was adjusted for the covariates included in Model 2 plus education, income, and occupation. Model 4 was additionally adjusted for creatinine clearance (CrCl), alanine aminotransferase (ALT) level, and comorbidities. Statistical significance was defined as *p* < 0.05. SAS (version 9.4; SAS Institute Inc., Cary, NC, USA) was used for all statistical analyses.

The institutional review boards of all investigational centers granted ethical approval for the study and informed consent was obtained from all subjects (Kyung Hee Medical Center IRB 1415–04). All methods were performed in accordance with relevant guidelines and regulations.

## Data Availability

The datasets generated during and/or analyzed during the current study are available from the corresponding author on reasonable request.
